# TRC: Mission Accomplished

**DOI:** 10.1289/ehp.115-a81

**Published:** 2007-02

**Authors:** Ernie Hood

Toxicogenomics is no longer in its infancy. The field is poised to make significant contributions to risk assessment, drug screening and development, clinical diagnosis and therapy, and policy decision making. That was the general consensus that emerged from the final meeting of the Toxicogenomics Research Consortium (TRC), a multicenter collaborative initiative established by the NIEHS in 2001 to serve as an extramural arm of the institute’s National Center for Toxicogenomics (NCT).

The conference, “Empowering Environmental Health Sciences Research with New Technologies,” was held 4–6 December 2006 in Chapel Hill, North Carolina, and was sponsored by the NIEHS, the University of North Carolina at Chapel Hill (UNC-CH) Center for Environmental Health and Susceptibility, the UNC-CH Lineberger Comprehensive Cancer Center, and Agilent Technologies, which manufactures microarrays along with other measurement tools. The meeting included presentations from grantees in two other recent efforts to support the development of applications of the “omics” technologies: the NIEHS Functional Proteomics Initiative, which was established in 2002, and the NIEHS/NIAAA Metabolomics Initiative, a consortium started in 2005 by the NIEHS and the National Institute on Alcohol Abuse and Alcoholism.

The conference began with a symposium recognizing the contributions to the field of toxicogenomics by the NCT and its director, Raymond Tennant. Tennant championed what has proven to be a vital concept in toxicogenomics: “phenotypic anchoring,” or the idea that the results of microarray experiments must be associated with particular phenotypes to be of informative value.

Richard Paules, a senior scientist in the NIEHS Laboratory of Molecular Toxicology, elaborates: “In order to understand the plethora of gene expression changes, where you’ve got five or ten thousand changes in a particular response [or] disease state, . . . it’s important to make a correlation, a link between a particular biological event and a group of gene expression changes. The linkage can then be tested, and subsequent experiments conducted in a hypothesis-driven manner.”

## A Refined Tool

The TRC was designed to last for five years, but is now in a sixth additional year with limited funding to wrap up outstanding projects. During its lifetime, the TRC has made tremendous strides, providing an infrastructure to validate the technology. “From the standpoint of where we started, when toxicogenomics was just a really neat tool, we’ve moved the tool forward,” says William Suk, director of the Center for Risk and Integrated Studies in the Division of Extramural Research and Training (CRIS/DERT) at the NIEHS. “It’s a tool that can now be used to address some very specific hypotheses . . . with regard to chemical structure and function.”

Toxicogenomics can now be considered a viable tool because for all intents and purposes the TRC, through a series of consortiumwide experiments, has accomplished one of its primary missions—standardization of microarray platforms and experimental methodologies. “We have achieved a high level of standardization in the technology,” says Tennant. “You can now use any of the commercial [microarray] platforms with confidence that you’re going to get a reliable, reproducible answer.”

One of the major achievements in the standardization process has been the development of statistical tools designed to control for inevitable variation in experimental conditions and methods. “We’re reaching a point where we understand where that variability is coming from and how to minimize it and control for it,” says conference co-organizer David Balshaw, a CRIS/DERT program administrator.

Ivan Rusyn, an assistant professor in the Department of Environmental Sciences and Engineering at UNC-CH, and a TRC grantee, agrees that standardization is a crucial step forward for toxicogenomics. “The task of standardization seems mundane and boring and not scientifically challenging,” he says, “but as toxicology is really applied science, we not only think of these new frontiers in science, but also how you can produce science that is [credible] to the public and to the regulator and can be used in some very practical applications.”

## Are the “Omics” Ready for Prime Time?

Meeting participants were cautiously optimistic that toxicogenomics, proteomics, and metabolomics are suitably advanced to begin to find application in policy decision making, in clinical medicine, and in broadening understanding of how gene–environment interactions can lead to human disease. According to Balshaw, we are entering “the age of systems biology,” with a growing ability to integrate data at different levels of organization and increasing levels of complexity.

“You can look at all twenty-one thousand genes simultaneously,” he says. “You can look at how the products of those genes—the proteins—are modified dynamically through phosphorylation and ubiquitination, and you can look at the products of those reactions—the small molecules in metabolomics. You can then begin to look at the integration of data at those three levels of biological organization to truly understand the mechanisms of environmentally induced disease.”

Tennant is confident that most of the predicted applications of toxicogenomics and the other technologies will come about, but cautions against unrealistic expectations of how long it will take. “I would say that plausibly within ten years, [toxicogenomics] could well be available in the clinic, as very targeted arrays and targeted platforms,” he says.

A test array for acetaminophen exposure, just one of several potentially toxic exposures studied by TRC scientists, may be one of the first applications to emerge from consortium experiments. “Our goal is to provide clinicians with a better tool to interpret liver injury with acetaminophen exposure, and a basis for making decisions on how to respond to overdose patients, many of whom come in borderline comatose,” says Paules. According to the American Association of Poison Control Centers, in 2003 more than 65,000 patients were seen in health care facilities for potential acetaminophen poisoning, and 327 died. “If we had a signature in the blood that would give insight as to whether an individual has been exposed to a severely toxic level and has suffered a severely toxic injury to the liver, it could help the clinician treat that patient,” Paules explains.

Genomics technologies are already having an impact in the pharmaceutical world, as evidenced by presentations at the meeting from Cynthia Afshari of Amgen and Weida Tong of the FDA. Amgen, a bio-therapeutics company, is using microarrays to screen candidate drug compounds for toxicity *in vitro*. The FDA recently issued guidelines to the drug industry to facilitate submission of genomic data, and is conducting its own standardization efforts, including an initiative called Microarray Quality Control.

## Onward

Although the TRC has run its course, there is still ample room for further development of the technologies, and there is still a great deal of knowledge to be gained from their implementation and continued refinement.

For example, TRC members at the Massachusetts Institute of Technology (MIT) have invented a rodent liver on a chip, known as a liver microbioreactor. It’s a three-dimensional physiological model of a rodent liver with all of the different liver cell types present, and it is responsive in the same way the whole organ in a whole animal would be. The device will allow testing of liver toxicity, which often involves multiple doses and multiple time points, to be conducted in a highly controlled high-throughput fashion. “It also lets you have a little bit cleaner system than the whole [animal], to test hypotheses about why things might be happening by adding and subtracting different cell types,” says Linda Griffith, a professor of biological and mechanical engineering at MIT.

The technology has emerged from the field of tissue engineering, where the focus is on growing replacement body parts. But according to Griffith, “there’s more of a push now to build replicas of human tissue to capture physiology in culture so that you can ultimately do high-throughput assays in human tissues—essentially to build a human body on a chip, to do predictive studies on how humans would respond.”

The next voyage of discovery for the field may well be yet another entry in the “omics” lexicon: epitoxicogenomics, or the application of toxicogenomics techniques to characterize DNA methylation patterns throughout the genome. “We see the product of methylation changes when we look at what genes are being turned on and turned off, [but] we often don’t know why they’re turned on and turned off,” explains Tennant. “If you can layer the methylation patterns on top of the expression patterns, the whole process of gene transcription will become much more understandable, and probably very predictable.”

The field will face other steep challenges in the years to come, such as developing the technologies and methodologies, particularly the computational and biostatistical tools, to characterize the effects of low-dose exposures and exposures to mixtures of chemicals. Either condition can increase by many times the difficulty of interpreting whole-genome assay results.

Several conference participants believe that incremental progress will be made in those areas as new, more sophisticated bioinformatics applications are formulated. On the issue of mixtures, Suk says, “The biostatistics and the informatics and the mathematical algorithms are there today to say, ‘this chemical does this,’ . . . [but] what we don’t have are the mathematical algorithms to help interpret these data.”

